# Recent advances in the progress of immune checkpoint inhibitors in the treatment of advanced gastric cancer: A review

**DOI:** 10.3389/fonc.2022.934249

**Published:** 2022-11-24

**Authors:** Jingjing Shen, Zhongming Wang

**Affiliations:** ^1^ School of Health Science and Engineering, University of Shanghai for Science and Technology, Shanghai, China; ^2^ Department of Radiation Oncology, Shidong Hospital, Yangpu District, Shidong Hospital Affiliated to University of Shanghai for Science and Technology, Shanghai, China

**Keywords:** gastric cancer, immune checkpoint inhibitors, chemotherapy, targeted therapy, radiotherapy

## Abstract

Most patients with advanced gastric cancer were treated with palliative therapy, which had a poor curative effect and a short survival time. In recent years, the clinical research of immune checkpoint inhibitors in advanced gastric cancer has made a breakthrough and has become an important treatment for advanced gastric cancer. The modes of immune checkpoint inhibitors in the treatment of advanced gastric cancer include single drug, combined chemotherapy, radiotherapy, and multiple immune drug combination therapy, among which combination therapy shows better clinical efficacy, and a large number of trials are currently exploring more effective combination therapy programs. In this paper, the new clinical research progress of immune checkpoint inhibitors in the treatment of advanced gastric cancer is reviewed, with an emphasis on combination therapy.

## Introduction

In 2020, more than one million people worldwide were diagnosed with gastric cancer, with an incidence rate of a fifth and a mortality rate of a fourth. Gastric cancer ranked second in the incidence and mortality of malignant tumors in China during the same period, and the morbidity and mortality of gastric cancer accounted for 43.9% and 48.6% of the global incidence and mortality, respectively (1). The diagnosis rate of early gastric cancer in China is less than 10%, and the vast majority of gastric cancer patients are already in the advanced stage when diagnosed, including locally advanced, distant metastasis, or recurrence (2). The 5-year survival rate of early gastric cancer can reach 70%. If distant metastasis occurs, the 5-year survival rate is only 6% (3). Advanced gastric cancer is mainly treated with palliative treatments, and the overall prognosis is poor. The median survival time after treatment is only 4–8 months (4, 5). Immunotherapy is an important treatment for gastric cancer; immune checkpoint inhibitors (ICIs) include cytotoxic T lymphocyte-associated antigen 4 (CTLA-4), programmed death 1 (PD-1), and programmed death ligand 1 (PD-L1), and more progress has been made in the clinical research of advanced gastric cancer. This paper reviews the clinical research on immune checkpoint inhibitors in the treatment of advanced gastric cancer in recent years to provide a theoretical basis for clinical practice and optimize the immunotherapy scheme of advanced gastric cancer.

## Immune checkpoint inhibitors monotherapy for advanced gastric cancer

The immune checkpoint is the regulator of the human immune system, preventing the inflammatory reaction of normal tissues caused by excessive activation of T cells. Tumor cells can abnormally express immune checkpoint molecules so that T cells cannot be activated, realize immune escape, and continue to grow. Immune checkpoint inhibitors improve the immune response to tumors by blocking the inhibitory signal of T-cell activation (6, 7). Pembrolizumab, nivolumab, avelumab, and other immune checkpoint inhibitors have made breakthroughs in the treatment of various types of tumors, including gastric cancer (GC). The initial treatment of unresectable/metastatic melanoma ipilimumab is also used to treat advanced gastric cancer (8).

### Anti-PD-1 antibody

The anti-PD-1 antibody mainly includes pembrolizumab, nivolumab, toripalimab, sintilimab, tislelizumab, etc.

A series of studies of KEYNOTE conducted an earlier clinical exploration of pembrolizumab.

KEYNOTE-012 evaluated the safety of pembrolizumab in the treatment of advanced gastric/gastroesophageal junction adenocarcinoma (GEJA) with positive PD-L1 (9). In total, 53% of patients had varying degrees of tumor reduction, and 13% had grades 3–4 treatment-related adverse events (TRAEs). KEYNOTE-059 explored the clinical efficacy of pembrolizumab in patients with disease progression after treatment. The objective response rate (ORR) was 11.6%, and the ORR of patients with PD-L1-positive and combined positive score (CPS) of ≥1 was 15.5%, which was significantly higher than the ORR of PD-L1-negative patients (6.4%). Grades 3–5 TRAEs occurred once or more in 17.8% of patients (10). Simultaneously, pembrolizumab first-line treatment of PD-L1-positive patients achieved better efficacy than posterior-line treatment (11).

Subsequently, three phase III randomized clinical trials of KEYNOTE-061, KEYNOTE-062, and KEYNOTE-063 were conducted to study the efficacy of pembrolizumab monotherapy in patients with advanced or metastatic gastroesophageal junction cancer (GEJC) (12–14).

In the KEYNOTE-061 study, the median survival time of patients with PD-L1 CPS ≥ 1 was 9.1 months in the pembrolizumab group and 8.3 months in the paclitaxel group (hazard ratio (HR), 0.82; unilateral *p* = 0.0421), which did not significantly improve the overall survival (OS) rate (12). However, pembrolizumab monotherapy was superior to paclitaxel in safety, with grades 3–5 TRAEs occurring in only 14% of patients compared to 35% in the paclitaxel group. In subsequent 2-year follow-up data published, pembrolizumab showed a trend of OS improvement compared with paclitaxel in patients with PD-L1 CPS ≥1, with a 2-year OS rate of 19.9% vs. 8.5% (15). KEYNOTE-063 was a trial in Asian populations (China, Malaysia, Korea), and results also suggested that pembrolizumab second-line treatment in Asian patients with PD-L1 CPS ≥1 G/GEJC was not superior to paclitaxel in OS (14).

In the KEYNOTE-062 trial, pembrolizumab monotherapy did not improve the prognosis of patients with advanced GC with PD-L1 CPS ≥1 compared with chemotherapy (13). However, in patients with PD-L1CPS ≥10, pembrolizumab monotherapy increased OS and reduced the risk of death by 31% compared with chemotherapy. Pembrolizumab monotherapy compared with chemotherapy had grades 3–5 TRAEs of 17% vs. 69%, and the safety was good. A subgroup analysis of Asian populations found significant OS benefits from pembrolizumab treatment (16). The median OS of patients with PD-L1 CPS ≥1 was 22.7 in the pembrolizumab group and 13.8 months in the chemotherapy group, and 28.1 and 14.8 months in patients with PD-L1 CPS ≥10, respectively. The risk of death in patients with PD-L1 CPS ≥1 and CPS ≥10 decreased by 46% and 57%, respectively. Compared with the posterior line therapy, pembrolizumab monotherapy can achieve a better therapeutic effect, but based on the whole series of KEYNOTE studies, it can be found that in the total population, the efficacy of pembrolizumab monotherapy was not significantly better or even lower than the standard treatment. However, there was an overall improved clinical therapeutic effect in patients with high expression of PD-L1, which showed that PD-L1 can be used as a biomarker for immunotherapy in patients with gastric cancer. Based on the above clinical outcomes, The Chinese Society of Clinical Oncology (CSCO): Clinical Guidelines for the Diagnosis and Treatment of Gastric Cancer (2021) only recommend pembrolizumab single-drug first-line therapy as a grade III recommendation (evidence 1B) for patients with PD-L1 CPS ≥1 (17).

Nivolumab is another anti-PD-1 antibody. In the ATTRACTION-2 trial, the median OS was 5.26 months in the nivolumab group and 4.14 months in the placebo group (HR, 0.63; *p* < 0.0001), and the ORR was 11% and 0, respectively (18). The incidence of grade 3–4 TRAEs in the nivolumab group was only 10%, with cutaneous and gastrointestinal adverse events. The risk of disease progression was also lower than that in the placebo group (HR, 0.60; *p* < 0.0001). The update of 2- and 3-year follow-up data also confirmed the long-term effect of nivolumab (19, 20). The long-term benefits of nivolumab were more pronounced in patients with complete response (CR) and partial response (PR), reducing the risk of death by 38%; the median OS was 26.28 months; and the 3-year OS rate was still as high as 35.5%. A cohort of CheckMate 032 also evaluated nivolumab in chemotherapy-refractory metastatic GC. The ORR of nivolumab monotherapy was 12%, the 1-year OS rate was 39%, the 1-year progression-free survival (PFS) rate was 8%, and the incidence of grade 3-4 TRAEs was 17%. The results were consistent with those of the ATTRACTION-2 trial (21).

Domestic clinical trials of ICIS for the treatment of GC are also ongoing. Toripalimab is the first domestically developed PD-1 inhibitor to be marketed in China. In a phase 1b trial in which toripalimab was used for chemotherapy-refractory advanced or metastatic gastric cancer, the ORR of toripalimab monotherapy was 12.1%, the median OS was 4.8 months, the median PFS was 1.9 months, and 22.4% of patients experienced grade 3 or more TRAEs, showing good antitumor activity (22). Domestic SHR-1210, also known as camrelizumab, is a high-avidity PD-1 inhibitor. In a phase I clinical study of advanced G/GEJC patients in China who had failed previous treatment, the efficacy of camrelizumab monotherapy was found to be promising. The ORR was 23.3%, and the median PFS was 8 weeks (23). However, the sample size of the trial was small, with only 30 patients. Based on these results, the efficacy of camrelizumab latter-line treatment in advanced GC is worthy of being studied in a larger cohort (**Table 1**).

**Table 1:** Clinical trials of PD-1 immune checkpoint inhibitors monotherapy for advanced gastric cancer.

Trial (reference)	Study design	Therapy line	Phase	Patient	Main finding			
					mOS (month)	mPFS (month)	ORR (%)	≥3TRAEs (%)
KEYNOTE-012 (9)	Pembrolizumab	≥1	I;b	PD-L1-positive G/GEJA	Asia: 11.4 Other: NR	Asia: 1.9 Other: 1.8	Asia: 24 Other: 21	13
KEYNOTE-059 (10)	Pembrolizumab	≥3	II	G/GEJC	5.6	2.0	11.6	17.8
KEYNOTE-061 (12)	Pembrolizumab vs. paclitaxel	2	III	G/GEJC	6.7 vs. 8.3	1.5 vs. 4.1	11.1 vs. 12.5	14 vs. 35
KEYNOTE-062 (13)	Pembrolizumab vs. FP/XP+ placebo	1	III	PD-L1-positive G/GEJC	10.6 vs. 11.1	2.0 vs. 6.4	14.8 vs. 37.2	17 vs. 69
KEYNOTE-063 (14)	Pembrolizumab vs. paclitaxel	2	III	Asian patients with PD-L1-positive G/GEJC	8 vs. 8	2 vs. 4	13 vs. 19	11 vs. 64
ATTRACTION-2 (18)	Nivolumab vs. placebo	≥3	III	Asian patients with G/GEJC	5.26 vs. 4.14	1.61 vs. 1.45	11 vs. 0	10 vs. 4
CheckMeta 032 (21)	Nivolumab	≥2	I;/II	G/GEJC, esophageal cancer	6.2	1.4	12	17
NCT02915432 (22)	Toripalimab	≥2	I;b/II	G/GEJA	4.8	1.9	12.1	22.4
NCT02742935 (23)	Camrelizumab (SHR1210)	≥2	I;	G/GEJA	NA	1.84	23.3	6.7

mOS, median overall survival; mPFS, median progression-free survival; ORR, objective response rate; TRAEs, treatment-related adverse events; PD-L1, programmed death ligand 1; G/GEJA, gastric or gastroesophageal junction adenocarcinoma; NR, not reached; G/GEJC, gastric and gastroesophageal junction cancer; FP, 5-fluorouracil+cisplatin; XP, capecitabine+cisplatin; CPS, combined positive score; NA, not applicable.

### Anti-PD-L1 antibody

Avelumab is a humanized anti-PD-L1 monoclonal antibody. A phase I multi-cohort trial was conducted to analyze the pharmacokinetics and immune activity, which proved the safety of avelumab in patients with solid tumors (24). However, in third-line treatments, compared with chemotherapy, avelumab monotherapy did not improve OS and PFS in patients with unresectable and locally advanced metastatic G/GEJC, but its safety was better than chemotherapy.

JAVELIN Gastric 300 study is a global phase III randomized trial. The median OS of avelumab and chemotherapy was 4.6 vs. 5.0 months, the median PFS was 1.4 and 2.7 months, and the ORR was 2.2% and 4.3%, respectively. Only safety was better than chemotherapy, with the incidence of grade 3 or higher TRAEs at 9.2% for avelumab and up to 31.6% for the chemotherapy group (25). The JAVELIN solid tumor trial found that patients without disease progression after first-line chemotherapy showed good clinical activity and safety with maintenance treatment with avelumab (26). To further evaluate strategies for first-line maintenance therapy, the JAVELIN Gastric 100 trial was conducted (27). The patients enrolled in the trial were untreated, advanced G/GEJC patients, and those who had not progressed after 12 weeks of oxaliplatin plus fluoropyrimidine first-line chemotherapy were randomly assigned to two groups: the continued chemotherapy group and the avelumab group. The median OS of the avelumab group and chemotherapy group was 10.4 and 10.9 months, respectively, and the 2-year OS rate was 22.1% vs. 15.5%. In patients with PD-L1 CPS ≥1, the median OS was 14.9 and 11.6 months in the avelumab and chemotherapy groups, respectively, and the incidence of grade 3 or higher TRAEs was 12.8% and 32.8%. The safety of avelumab was superior to chemotherapy, but the second-line treatment did not bring better survival benefits in all patients with advanced metastatic G/GEJC or designated PD-L1-positive patients. Several studies have shown that in the first-line maintenance and second-line and third-line treatment, avelumab did not bring survival benefits to patients, but it was safer than chemotherapy and had the potential for late maintenance therapy.

Envafolimab is the world’s first subcutaneous injection of a PD-L1 inhibitor. The first-in-human phase I trial was conducted in patients with advanced solid tumors, showing good safety and antitumor activity (28). In a phase II trial, envafolimab monotherapy was studied in patients with previously treated Chinese advanced defective mismatch repair (dMMR) or microsatellite instability-high (MSI-H) solid tumors, including gastric cancer. In the total population, ORR was 42.7%, DCR was 66.0%, median PFS was 11.1 months, which did not reach the median OS, and the 1-year OS rate was 74.6%. In patients with advanced GC, the ORR was 44.4%. In terms of safety, the incidence of grade 3–4 TRAEs was only 16%. Envafolimab monotherapy has shown significant efficacy and good safety in advanced dMMR/MSI-H solid tumor patients who have failed at least first-line treatment (29). Based on this trial, envafolimab was approved in China for the treatment of adult patients with unresectable or metastatic dMMR/MSI-H advanced solid tumors and is the first and only subcutaneous injection PD-L1 inhibitor approved for marketing in the world (30).

### Anti-CTLA-4 antibody

Ipilimumab is a humanized anti-CTLA-4 antibody that blocks the interaction between CTLA-4 and its ligand and promotes T-cell activation to inhibit the tumor response. However, the clinical efficacy of ipilimumab monotherapy for advanced GC is not as good as the best supportive care (BSC) (31). Advanced metastatic G/GEJC patients with stable conditions after first-line chemotherapy were randomized to ipilimumab at 10 mg/kg every 3 weeks for four times and then to 10 mg/kg every 12 weeks for 3 years or BSC with immune-related PFS as the primary endpoint. The study did not meet its primary endpoint because ipilimumab did not improve immune-related PFS, resulting in trial cessation (2.92 vs. 4.9 months). The 1-year PFS rate of ipilimumab was 8.4% and BSC was 16.1%, the median OS was 12.7 and 12.1 months, respectively. Patients treated with ipilimumab had a significantly lower ORR than those treated with BSC (1.8% vs. 7.0%) and a higher incidence of grade 3–4 TRAEs than those treated with BSC (22.8% vs. 8.9%). Ipilimumab was not suitable for monotherapy in patients with advanced gastric cancer, which not only did not increase the clinical benefits of patients but also produced a higher incidence of adverse reactions.

## ICIs combined with other treatments of advanced GC

### Combined with chemotherapy

The median PFS and OS of KEYNOTE-059 cohort 2 pembrolizumab combined with first-line chemotherapy in patients with advanced G/GEJA were 6.6 and 13.8 months, respectively. The incidence of TRAEs was higher at 76.0% (11). In the KEYNOTE-062 trial, pembrolizumab plus first-line chemotherapy did not outperform chemotherapy in patients with advanced G/GEJC, and there was no clinical benefit (13).

In the phase II ATTRACTION-4 trial, nivolumab was combined with S-1: tegafur–gimeracil–oteracil potassium+oxaliplatin (SOX) or capecitabine+oxaliplatin (CAPOX) in the first-line treatment of advanced G/GEJC patients. The ORR was 57.1% and 76.5% respectively, and the disease control rate (DCR) was 81.0% and 88.2% respectively, showing excellent clinical efficacy (32). ATTRACTION-4 Phase III trial further verified the results of the phase II trial. The ORR of nivolumab in combination with chemotherapy (SOX or CAPOX) or placebo plus chemotherapy was 57.5% and 47.8% (*p* = 0.0088), respectively, effectively improving the response rate. The median PFS was 10.45 and 8.34 months, respectively (HR, 0.68; *p* = 0.0007). Nivolumab plus chemotherapy significantly prolonged PFS, but the difference in survival was not significant (OS was 17.45 vs. 17.15 months). Although nivolumab plus chemotherapy had a higher incidence of TRAEs than chemotherapy (18% vs. 9% for grade 3–4 serious adverse events), the safety profile was still manageable (33). The ATTRACTION-4 trial was based on Asian populations and demonstrated the superior efficacy and safety of nivolumab in combination with first-line chemotherapy, providing a new approach to first-line immunotherapy for patients with unresectable advanced, recurrent, or metastatic GC in China.

CheckMate 649 showed that in patients with PD-L1 CPS ≥5, nivolumab combined with chemotherapy reduced the risk of death by 29% compared with chemotherapy, with a median OS improvement of 3.3 months (14.4 vs. 11.1 months; HR, 0.71; *p* < 0.0001). The 1-year OS rate was 57% and 46%, respectively. In both PD-L1 CPS ≥1 and the overall population, nivolumab combined with chemotherapy had better OS and PFS than chemotherapy, with 1-year OS rates of 56% and 47%, and 1-year PFS rates of 34% and 22%, respectively, consistent with CPS ≥5. In terms of safety, the most common TRAEs at any level were similar to those reported in previous trials, with subgroups and the population generally consistent (34). In the Chinese subgroup analysis, nivolumab combined with chemotherapy showed better clinical efficacy than the overall population (35). In the untreated Chinese population, the median OS for nivolumab in combination with chemotherapy and chemotherapy alone was 14.3 and 10.3 months (HR, 0.61), and the median PFS was 8.3 and 5.6 months, respectively. Clinical benefits were significant, and no immune-related deaths occurred. In the population with PD-L1 CPS ≥5, the survival benefit of nivolumab combined with chemotherapy was more obvious. The ORR was 63% and 43%, respectively, and the OS was 15.5 vs. 9.6 months (HR, 0.54). OS increased by nearly 6 months, reducing the risk of death by 46%, significantly better than 29% of the overall population. Nivolumab in combination with chemotherapy had twice the PFS of chemotherapy alone (median PFS, 8.5 vs. 4.3 months; HR, 0.52). Survival benefits were also significant in patients with PD-L1 CPS ≥1, with median OS of 14.3 and 9.9 months (HR, 0.62), respectively. Overall analysis showed that nivolumab combined with chemotherapy in the first-line treatment of HER2-negative advanced G/GEJC/esophageal adenocarcinoma was not limited by PD-L1 expression and benefited the whole population. The CheckMate649 trial is the first breakthrough in gastric cancer immunotherapy in nearly a decade, establishing a new immunotherapy standard for first-line treatment of PD-L1 CPS ≥5, HER2-negative metastatic gastric cancer. Based on the results of the CheckMate 649 and ATTRACTION-4 trials, the 2021 CSCO Clinical Guidelines for the Diagnosis and Treatment Of Gastric Cancer listed nivolumab combined with chemotherapy for first-line treatment of HER2-negative advanced gastric cancer (CPS ≥5) as a grade I recommendation (evidence 1A) (17).

Sintilimab is a recombinant humanized anti-PD-1 monoclonal antibody with a higher avidity for PD-1 than nivolumab and pembrolizumab and consistently and effectively blocks the PD-1 signaling pathway (36). Sintilimab combined with chemotherapy has also achieved excellent results in the treatment of advanced G/GEJA.ORR was 85%, DCR was 100%, and the incidence of grade 3–4 TRAEs was 55% in studies with sintilimab combined with CapeOX in first-line treatment of advanced G/GEJA patients; the most common TRAE was decreased platelet count, accounting for 45% of cases, showing excellent clinical efficacy and controlled safety (37). ORIENT-16 was a large randomized, double-blind phase III trial that found that sintilimab combined with first-line chemotherapy significantly reduced the risk of death in patients with advanced G/GEJA, with CPS ≥5 (HR, 0.660) and in the total population (HR, 0.766) (38). In patients with CPS ≥5, the median OS of sintilimab combined with CapeOX and the control group was 18.4 and 12.9 months, respectively, with a survival benefit of 5.5 months. In the total population, the median OS was 15.2 and 12.3 months, respectively, and the median OS increased by 2.9 months. In CPS ≥5 and the total population, the PFS of sintilimab combined chemotherapy was better than that of the control group (HR was 0.628 and 0.636). The results of this study demonstrated that sintilimab in combination with chemotherapy in first-line treatment of Chinese patients with advanced G/GEJA has excellent clinical efficacy and controllable safety, regardless of PD-L1 expression. Another trial of sintilimab combined with Nab-paclitaxel for second-line treatment of advanced G/GEJA patients showed an ORR of 41.9%, DCR of 83.9%, and a median PFS of 5.2 months, showing good antitumor activity and controlled safety, which is still in progress (39).

The ORR and DCR of toripalimab combined with oxaliplatin+capecitabine (XELOX) in the latter-line treatment of advanced GC were 66.7% and 88.9%, showing excellent antitumor activity. The incidence of TRAEs above grade 3 was 38.9%, and the safety was also manageable (22). In the first-line treatment of advanced G/GEJC patients with toripalimab combined with SOX, the ORR of patients who completed two cycles of treatment was 57%, DCR was 93%, and the ORR of patients who completed four cycles of treatment was 60%; DCR was 90%. They showed good antitumor activity, and most of the TRAEs were grades 1–2. The trial is still in progress (40).

The phase II clinical study of tislelizumab combined with chemotherapy in the treatment of advanced esophageal squamous cell carcinoma and G/GEJA showed no serious adverse reactions. In general, it was safe and well tolerated. ORR and DCR were 46.7% and 80%, respectively. The median PFS was 6.1 months, and the 1-year OS rate was 62%, which had not reached the median OS (41). Subsequently, a large randomized, double-blind, placebo-controlled phase III trial was conducted to study the efficacy and safety of tislelizumab combined with first-line chemotherapy in patients with advanced G/GEJA, and the trial is ongoing (42).

Envafolimab in combination with 5-fluorouracil, leucovorin, and oxaliplatin (FOLFOX) has shown superior clinical efficacy and controlled safety in untreated patients with advanced G/GEJC. The incidence of grade 3–4 TRAEs was 73.3%, mostly with neutrophil count decreased (46.7%), anemia (20%), etc. The safety was controllable, the ORR was 60%, and the median PFS was 6.8 months (43).

AK104 is a novel tetramer form of recombinant humanized PD-1/CTLA-4 bispecific antibody, which can bind to both PD-1 and CTLA-4 at the same time and has a higher avidity. In patients with advanced G/GEJA, AK104 in combination with XELOX showed superior antitumor activity and a controllable safety profile, regardless of PD-L1 expression, without grade 4–5 TRAEs. The 6-month PFS rate of AK104 at 4 mg/kg + XELOX was 69.5%, and the trial is still in progress (44) (**Table 2**).

**Table 2:** Clinical trials of immune checkpoint inhibitors combined with chemotherapy in the treatment of advanced gastric cancer.

Target	Trial (reference)	Therapy line	Phase	Study design	Patient	Main finding			
						mOS (month)	mPFS (month)	ORR (%)	≥3TRAEs (%)
PD-1	KEYNOTE-059 (11)	1	II	Pembrolizumab+cisplatin+5-fluorouracil/capecitabine vs. pembrolizumab	G/GEJA	13.8 vs. 20.7	6.6 vs. 3.3	60 vs. 25.8	76 vs. 22.6
	KEYNOTE-062 (13)	1	III	Pembrolizumab+chemotherapy (cisplatin+fluorouracil/capecitabine) vs. placebo+chemotherapy	PD-L1-positive G/GEJC	12.5 vs. 11.1	6.9 vs. 6.4	48.6 vs. 37.2	73 vs. 69
	ATTRACTION-4 (32)	1	II	Nivolumab+SOX vs. nivolumab+CAPOX	Asian patients with HER2-negative G/GEJC	NR	9.7 vs. 10.6	57.1 vs. 76.5	57.1 vs. 66.7
	ATTRACTION-4 (33)	1	III	Nivolumab+chemotherapy (S-1/capecitabine+oxaliplatin) vs. placebo+chemotherapy	Asian patients with HER2-negative G/GEJC	17.45 vs. 17.15	10.45 vs. 8.34	57.5 vs. 47.8	58 vs. 50
	CheckMate 649 (34)	1	III	Nivolumab+chemotherapy (CAPOX or FOLFOX) vs. chemotherapy	HER2-negative G/GEJC or oesophageal adenocarcinoma	13.8 vs. 11.6	7.7. vs. 6.9	60 vs. 45	60 vs. 44
	NCT02937116 (37)	1	I;b	Sintilimab+oxaliplatin/capecitabine	G/GEJA	NR	7.5	85	55
PD-1	ORIENT-16 (38)	1	III	Sintilimab+CAPOX vs. placebo+CAPOX	G/GEJA	15.2 vs. 12.3	NA	65.1 vs. 58.7	59.8 vs. 52.5
	NCT04140318 (39)	2	II	Sintilimab+Nab-paclitaxel	G/GEJA	NA	5.2	41.9	45.2
	NCT02915432 (22)	1	I;b/II	Toripalimab+CAPOX	G/GEJA	NR	5.8	66.7	38.9
	NCT04202484 (40)	1	NA	Toripalimab+SOX	G/GEJC	NA	NA	57/60/43	NA
	NCT03469557 (41)	1	II	Tislelizumab+CAPOX	ESCC or G/GEJA	NR	6.1	46.7	66.7
	NCT03777657 (42)	1	III	Tislelizumab+platinum/fluoropyrimidine vs. placebo+platinum/fluoropyrimidine	G/GEJA	NA	NA	NA	NA
PD-L1	CTR20181124 (43)	1	II	Envafolimab+FOLFOX	G/GEJC	NA	6.8	60	73.3
PD-1/CTLA-4	NCT03852251 (44)	1	I;b	AK104+CAPOX	G/GEJA	NA	NA	66.7	29.4

mOS, median overall survival; mPFS, median progression-free survival; ORR, objective response rate; TRAEs, treatment-related adverse events; PD-1, programmed death 1; G/GEJA, gastric or gastrooesophageal junction adenocarcinoma; G/GEJC, gastric and gastroesophageal junction cancer; S-1, tegafur–gimeracil–oteracil; SOX, S-1+oxaliplatin; CAPOX, capecitabine+oxaliplatin; NR, not reached; NA, not applicable; PD-L1, programmed death ligand 1; CPS, combined positive score; ESCC, esophageal squamous cell carcinoma; FOLFOX, 5-fluorouracil+leucovorin+oxaliplatin.

### Combined with targeted therapy

The population that would benefit from targeted therapy alone for advanced gastric cancer is small. Due to the good clinical efficacy of ICIs in the treatment of advanced GC, many scholars began to study ICIs combined with targeted therapy. Currently, human epidermal growth factor receptor 2 (HER2) and vascular epidermal growth factor (VEGF) are the most studied targets.

#### Combined with HER2

HER2 is a transmembrane tyrosine kinase receptor, and its overexpression is related to the pathological mechanisms and poor prognosis of breast cancer, gastric cancer, and other tumors (45). Trastuzumab is an anti-HER2 monoclonal antibody. The median OS of patients with HER2-positive metastatic G/GEJC treated with trastuzumab plus fluorouracil and platinum can reach 13.8 months (46). In a phase II trial pembrolizumab in combination with trastuzumab and first-line chemotherapy in patients with HER2-positive metastatic GC, median PFS was 13 months, the 6-month PFS rate was 70%, the median OS was as high as 27.3 months, the 1-year OS rate was 80%, and the DCR was 100% (47). The PANTHERA trial also studied the triple combination therapy of pembrolizumab, trastuzumab, and cisplatin plus capecitabine in first-line treatment of patients with HER2-positive metastatic GC, with an ORR of 76.7%, median PFS of 8.6 months, median OS of 19.3 months, and DCR of 97.7% (48, 49).

The above two trials were small-sample, single-arm trials. KEYNOTE-811 was a global, randomized, double-blind, placebo-controlled phase III trial in which intermediate data showed an ORR of 74.4% for pembrolizumab plus trastuzumab and chemotherapy, with a complete response rate of 11.3% in the trial group, nearly four times that of the control group (3.1%). The incidence of grade 3–5 TRAEs was similar in both groups: 57.1% in the trial group vs. 57.4% in the control group (50). Pembrolizumab plus trastuzumab and chemotherapy significantly increased patient survival benefits.

Pembrolizumab in combination with margetuximab, an Fc-engineered, anti-HER2 monoclonal antibody, showed acceptable safety in advanced HER2-positive gastroesophageal adenocarcinoma after being treated with previous trastuzumab combined with chemotherapy, and the incidence of grade 3–5 TRAEs were 20%. The median OS was 12.5 months, the median PFS was 2.7 months, the ORR was 18.48%, and the DCR was 53% (51). Pembrolizumab in combination with margetuximab is expected to provide chemotherapy-free treatment for HER2-positive patients. A MAHOGANY study of ICIs combined with margetuximab ± chemotherapy in the first-line treatment of HER2-positive advanced GC/GEJA is underway (52). The combination of KN026 (HER2-targeted bispecific antibody) and KN046 (anti-CTLA-4/PD-L1 bispecific antibody) independently developed in China in the first-line treatment of patients with advanced HER2-positive GC had an ORR of up to 86% (53). The phase II trial for efficacy and safety verification of KN026 combined with KN046 in the treatment of HER2-positive and advanced solid tumors is ongoing, and we look forward to the announcement of the results.

#### Combined with VEGF

Blocking VEGF or vascular epidermal growth factor receptor 2 (VEGFR2) pathway can destroy tumor microvascularity and inhibit tumor growth, normalize abnormal tumor vascularity, and increase tumor oxygenation and drug supply (54). At present, ramucirumab, lenvatinib, regorafenib, and apatinib are widely used as anti-VEGF or VEGFR2 monoclonal antibodies.

In patients with advanced G/GEJA who received pembrolizumab in combination with ramucirumab as first-line therapy, 64% were grade 3 TRAEs, without grade 4 or 5 AEs, and the safety was controllable. Median OS was 14.6 months, the median PFS was 5.6 months, and the ORR was 25%. Among patients with PD-L1 positivity, the clinical benefit was superior, with a median OS of 17.3 months, a median PFS of 8.6 months, and an ORR of 32% (55). The results of this trial provide clinical evidence for the combination of anti-VEGF or anti-VEGFR2 antibodies and PD-1 inhibitors in the treatment of patients with metastatic G/GEJA. Pembrolizumab combined with lenvatinib in first-line and latter-line treatment of advanced G/GEJA had an ORR of 69%, a median PFS of 7.1 months, no TRAEs of grade 4 or higher, a manageable safety, and demonstrated superior clinical effects (56). The above two trials are single-arm and small-sample trials, and it is necessary to conduct confirmatory studies in larger cohorts.

REGONIVO is a phase 1B clinical trial designed to evaluate nivolumab in combination with regorafenib in previously treated patients with advanced GC and colorectal cancer. In the GC cohort, the median PFS was 5.6 months and the median OS was 12.3 months; the ORR was 44%. The ORR of PD-L1 CPS ≥1 was higher than that of the general population (60%), which was better than nivolumab monotherapy in patients with metastatic GC (57). Based on the efficacy and safety evaluation, regorafenib at 80 mg is the optimal dose for combination therapy with nivolumab. Nivolumab in combination with paclitaxel and ramucirumab also showed good efficacy in the second-line treatment of advanced GC, with a median OS of 13.1 months, a 1-year OS rate of 55.8%, and a 6-month PFS rate of 46.5%. The median OS of patients with CPS ≥1 was higher than that of patients with CPS <1 (13.8 vs. 8.0 months), indicating controllable safety (58).

Clinical trials of immunotherapy combined with targeted therapy are also being carried out in China, and the trials of camrelizumab combined with apatinib in the first-line, second-line, and latter-line treatment of metastatic GC have reported the results successively. The toxicity of camrelizumab combined with apatinib in the treatment of advanced GC was controllable; 95.3% of patients were in stage IV at the time of enrollment. ORR was 16%, median PFS was 2.9 months, and median OS was 11.4 months (59). In the phase II trial of second-line treatment of patients with advanced G/GEJA treated with camrelizumab plus apatinib plus S-1, the ORR was 29.2%, and the median PFS was 6.5 months. Only 25% of patients experienced grade 3–4 TRAEs, and no severe TRAEs or related deaths occurred. Good efficacy and safety have been shown in advanced GC patients with first-line refractory treatment (60). In patients with untreated advanced GC, patients without disease progression after four to six cycles of camrelizumab plus CAPOX are treated with camrelizumab plus apatinib until disease progression or intolerable toxicity. The ORR was 58.3%. It was similar to the ORR (57.9%) of pembrolizumab combined with cisplatin and 5-fluorouracil in KEYNOTE-059 and the ORR (57.5%) of nivolumab combined with SOX in ATTRACTION-4 (11, 33). In total, 2.1% of patients had CR, the DCR was 93.8%, the median OS was 14.9 months, the median PFS was 6.8 months, and the incidence of grade 3 or higher TRAEs were 68.6% (61). This study is the first trial of sequential ICIs combined with targeted therapy for advanced GC after ICIs combined with chemotherapy, and the results are encouraging. Durvalumab, an anti-PD-L1 monoclonal antibody, combined with ramucirumab therapy, had an ORR of 21%, median OS and PFS of 12.4 and 2.6 months, respectively, in pretreated advanced GC, and the incidence of grade 3 or higher TRAEs were 32.1%. It has good antitumor activity and safety (62) (**Table 3**).

**Table 3:** Clinical trials of immune checkpoint inhibitors combined with targeted therapy for advanced gastric cancer.

Target	Trial (reference)	Therapy line	Phase	Study design	Patient	Main finding			
						mOS (month)	mPFS (month)	ORR (%)	≥3TRAEs (%)
PD-1 HER2	NCT02954536 (47)	1	II	Pembrolizumab+trastuzumab+FP/CAPOX	HER2-positive esophagogastric cancer	27.3	13	91	57
	PANTHERA (48, 49)	1	I;b/II	Pembrolizumab+trastuzumab+XP	HER2-positive GC	19.3	8.6	76.7	74.7
	KEYNOTE-811 (50)	1	III	Pembrolizumab+trastuzumab+FP/CAPOX vs. placebo+trastuzumab+FP/CAPOX	HER2-positive G/GEJA	NA	NA	74.4 vs. 51.9	57.1 vs. 57.4
	CP-MGAH22–05 (51)	≥2	I;b/II	Pembrolizumab+margetuximab	HER2-positive, gastroesophageal adenocarcinoma	12.5	2.7	18.48	20
PD-1 LAG-3 HER2	MAHOGANY (52)	1	II/III	Retifanlimab+margetuximab; trastuzumab+CAPOX/FOLFOX-6 vs. margetuximab ± tebotelimab/retifanlimab+CAPOX/FOLFOX-6	HER2-positive G/GEJA or esophageal adenocarcinoma	NA	NA	NA	NA
CTLA-4 PD-L1 HER2	NCT04040699 (53)	1	I;b	KN026+KN046	HER2-positive G/GEJC	NA	NA	86	NA
PD-1 VEGFR2	NCT02443324 (55)	1	I;a/b	Ramucirumab+pembrolizumab	GC/GEJC	14.6	5.6	25	64
PD-1 VEGF	EPOC1706 (56)	1/2	II	Pembrolizumab+lenvatinib	G/GEJA	NR	7.1	69	48
PD-1 VEGFR2	REGONIVO (57)	≥3	I;b	Nivolumab+regorafenib	GC	12.3	5.6	44	40 (all)
	UMIN000025947 (58)	2	I;/II	Nivolumab+paclitaxel+ramucirumab	GC/GA	13.1	5.1	37.2	90.7
	NCT03463876 (59)	≥2	I;a/b	SHR-1210+apatinib	GC/GEJC	11.4	2.9	16	60 (all)
	NCT04345783 (60)	2	II	Camrelizumab (SHR1210)+apatinib+S-1	G/GEJA	NR	6.5	29.2	25
	NCT03472365 (61)	1	II	Camrelizumab+CAPOX; camrelizumab+apatinib	VEGFR2-negative G/GEJA	14.9	6.8	58.3	68.8
PD-L1 VEGFR2	NCT02572687 (62)	≥2	I;a/b	Durvalumab+ramucirumab	G/GEJA	12.4	2.6	21	32.1

PD-1, programmed death 1; HER2, human epidermal growth factor receptor 2; FP, 5-fluorouracil+cisplatin; CAPOX, capecitabine+xaliplatin; XP, capecitabine+cisplatin; GC, gastric cancer; G/GEJA, gastric or gastrooesophageal junction adenocarcinoma; FOLFOX-6, oxaliplatin+5-fluorouracil+leucovorin; VEGFR2, vascular epidermal growth factor receptor; GC/GEJC, gastric cancer/gastroesophageal junction cancer; VEGF, vascular epidermal growth factor; GA, gastric adenocarcinoma; S-1, tegafur–gimeracil–oteracil; CAPOX, capecitabine+oxaliplatin; NR, not reached; NA, not applicable.

### Combined with immunotherapy

The CheckMate 032 trial randomly assigned all patients into three groups: nivolumab 3 mg/kg (NIVO3), nivolumab 1 mg/kg combined with ipilimumab 3 mg/kg (NIVO1+IPI3), and nivolumab 3 mg/kg combined with ipilimumab 1 mg/kg (NIVO3+IPI1); 79% of patients had received second or more lines of treatment. The ORR for NIVO1+IPI3 was 24% and 8% for NIVO3+IPI1, the median OS was 6.9 and 4.8 months, respectively, and the 1.5-year OS rate was 28% and 13%, respectively. NIVO1+IPI3 showed a lasting survival benefit. The median PFS was similar in both groups (1.4 vs. 1.6, respectively), but the half-year PFS rate of NIVO1+IPI3 was twice that of NIVO3+IPI1 (24% vs. 12%). In terms of safety, the incidence of NIVO1+IPI3 grade 3–4 TRAEs was higher than that of NIVO3+IPI1 (47% vs. 27%) (21). Nivolumab in combination with ipilimumab has shown clinically significant antitumor activity and lasting benefit in patients with advanced GC.

Due to serious adverse events during the trial, enrollment in the CheckMate 649 trial’s dual immunization cohort NIVO1+IPI3 has been closed (34, 63). In all randomized populations, the median OS for NIVO1+IPI3 was 11.7 and 11.8 months in the chemotherapy group. The incidence of grade 3–4 TRAEs was 38%, and the safety was better than that of the chemotherapy group (46%) and nivolumab combined with chemotherapy (60%). Among patients with PD-L1 CPS ≥5, the median OS was 11.2 months compared with 11.6 months in the chemotherapy group. The ORR was 27%, much lower than the chemotherapy group (47%) and the nivolumab combination group (60%). Nivolumab in combination with ipilimumab did not increase survival benefits compared to chemotherapy alone or nivolumab in combination with chemotherapy.

Durvalumab combined with tremelimumab (an anti-CTLA-4 antibody) for the second-line or latter-line treatment of metastatic or recurrent G/GEJA had a low response rate in both monotherapy and combination therapy. The ORR of the second-line and third-line combination therapies was 7.4% and 4.0%, respectively, but the 1-year OS rate was 37% and 38.8%, and the incidence of grade 3–4 TRAEs was also low, at 17% and 16%, respectively (64).

### Combined with radiotherapy

Radiotherapy can activate the immune response, but it also produces the abscopal effect of reducing the lesions outside the irradiation target. Radiotherapy combined with PD-1/PD-L1 inhibitors has a better synergistic effect (65–67). A retrospective study found that patients with metastatic GC who had previously received anti-PD-1 therapy were sensitized to palliative radiotherapy in subsequent treatment. Immunoassay also showed that anti-PD-1 therapy can enhance the efficacy of radiotherapy through immune activation (68). The CheckMate577 trial looked at nivolumab in patients with esophageal cancer, or GEJC, who had previously undergone surgery following neoadjuvant radiotherapy and chemotherapy. In total, 794 patients were randomized 2:1 to nivolumab or placebo. The median PFS in the two groups were 22.4 vs. 11.0 months (HR, 0.69; 96.4% CI, 0.56–0.86; *p* < 0.001), indicating that nivolumab significantly extended disease-free survival after neoadjuvant chemoradiotherapy. Median distant metastasis-free survival was 28.3 vs. 17.6 months (HR 0.74), and nivolumab reduced the risk of distant recurrence by 26% (69).

In a trial of camrelizumab combined with chemoradiotherapy for locally advanced gastric adenocarcinoma, patients were first treated with induction chemotherapy for 3 weeks and received XELOX regimen: capecitabine 1,000 mg/m^2^, twice a day for 2 weeks, and oxaliplatin 130 mg/m^2^ on day 1. Concurrent chemoradiotherapy was started within 1 week after induction chemotherapy for 5 weeks: intensity-modulated radiotherapy was given for tumors and high-risk lymphatic drainage areas; total dose: 45 Gy/25 F, 1.8 Gy/F; oral capecitabine: 850 mg/m^2^, twice a day. Two to three weeks after concurrent chemoradiotherapy, one cycle of XELOX regimen consolidation chemotherapy was performed. Camrelizumab (200 mg) was injected intravenously every 3 weeks from the beginning of induction chemotherapy until 3 weeks before surgery. Patients were required to receive four cycles of XELOX adjuvant chemotherapy within 4–6 weeks postoperatively. Results indicated that camrelizumab combined with radiation and chemotherapy was an effective treatment for patients with locally advanced GC, ORR was 93.3%, the pathological complete response (pCR) rate was 33.3%, the primary endpoint of the phase I trial, and the phase II trial was ongoing (70, 71). Neoadjuvant concurrent chemoradiotherapy followed by surgery in patients with locally advanced esophageal, or GEJA, who received durvalumab showed an excellent survival benefit with 2-year recurrence-free survival and OS of 55.5% and 67.4%, respectively (72). Concurrent chemoradiotherapy with sintilimab (200 mg, once every 3 weeks) had also achieved good clinical results in the treatment of locally advanced G/GEJA (73). Induction chemotherapy (S-1+Nab-paclitaxel) was given first, followed by concurrent radiotherapy (45 Gy/25 F, 1.8 Gy/F), and finally consolidation chemotherapy (S-1+Nab-paclitaxel). In total, 42.1% of patients achieved pCR, and the incidence of grade 3–4 TRAEs was 39.3%. The trial is ongoing.

Currently, more clinical trials of ICIs in combination with radiotherapy for GC are underway, such as NCT03044613, NCT03064490, NCT03490292, etc. More prospective studies are expected to determine the mechanism of action of radiotherapy for GC and the combined ICI regimen.

## Conclusion

Immune checkpoint inhibitors have achieved good results in clinical studies of advanced gastric cancer and have been recommended from third-line to second-line to first-line treatment, from grade III to grade II to grade I;, changing the mode of gastric cancer treatment. Several clinical trials have explored the clinical effects of monotherapy with immune checkpoint inhibitors, combined chemotherapy, radiotherapy, and targeted therapy, and the results showed that immunotherapy combined with other therapies had obvious clinical advantages. However, more clinical studies are needed on the drug administration order combination regimen and benefit groups of combination therapy. The incidence of gastric cancer in China accounts for nearly half of the global incidence of gastric cancer; however, gastric cancer heterogeneity, complex tumor microenvironment, pathogenesis, and therapy of Chinese and Western are different. In China, patients with gastric cancer should be encouraged to actively participate in clinical research to develop an immune treatment for advanced gastric cancer and provide more reliable clinical evidence.

## Author contributions

JS and ZW drafted the paper and contributed to the writing and critical review of the manuscript. All authors contributed to the article and approved the submitted version.

## Conflict of interest

The authors declare that the research was conducted in the absence of any commercial or financial relationships that could be constructed as a potential conflict of interest.

## Publisher’s note

All claims expressed in this article are solely those of the authors and do not necessarily represent those of their affiliated organizations, or those of the publisher, the editors and the reviewers. Any product that may be evaluated in this article, or claim that may be made by its manufacturer, is not guaranteed or endorsed by the publisher.
